# Environmental Health Is Overlooked in Longevity Research

**DOI:** 10.3390/antiox14040421

**Published:** 2025-03-31

**Authors:** Robin Mesnage

**Affiliations:** 1 Department of Nutritional Sciences, School of Life Course Sciences, Faculty of Life Sciences and Medicine, King’s College London, London SE1 9NH, UK; robin.mesnage@kcl.ac.uk; 2 Buchinger Wilhelmi Clinic, Wilhelm-Beck-Straße 27, 88662 Überlingen, Germany

**Keywords:** longevity, environmental health, aging

## Abstract

Aging is a multifactorial process influenced by genetic predisposition and lifestyle choices. Environmental exposures are too often overlooked. Environmental pollutants—ranging from airborne particulate matter and heavy metals to endocrine disruptors and microplastics—accelerate biological aging. Oxidative stress is a major molecular initiating event, driving inflammation and toxicity across biological levels. We detail the mechanisms by which pollutants enhance reactive oxygen species (ROS) production. This oxidative stress inflicts damage on DNA, proteins, and lipids, accelerating telomere shortening, dysregulating autophagy, and ultimately driving epigenetic age acceleration. For instance, exposure to polycyclic aromatic hydrocarbons, benzene, and pesticides has been associated with increased DNA methylation age. Early-life exposures and lifestyle factors such as tobacco and alcohol consumption further contribute to accelerated biological aging. The cumulative loss of healthy life years caused by these factors can conceivably reach between 5 and 10 years per person. Addressing pollutant-induced accelerated aging through regulatory measures, lifestyle changes, and therapeutic interventions is essential to mitigate their detrimental impacts, ultimately extending healthspan and improving quality of life in aging populations.

## 1. Introduction

Aging is a progressive loss of physiological integrity, leading to impaired function and increased vulnerability to death [1]. It is influenced by genetic predisposition, lifestyle choices, and environmental factors [2]. The pursuit of extending human lifespan has been a longstanding objective in scientific research. Advances in genetics, regenerative medicine, and metabolic interventions have shown promise in slowing aging and prolonging life [3]. However, simply increasing lifespan without ensuring a parallel improvement in healthspan—the period of life spent free from chronic disease—may offer limited societal benefits [4].

Contributors to a reduction in healthspan are already well identified. Unhealthy dietary habits contribute to approximately one in five deaths worldwide [5]. Adopting a healthy lifestyle, including a balanced diet, regular physical activity, avoiding smoking, and moderating alcohol consumption, has been linked to a substantial increase in life expectancy—up to 14 years in women and 12 years in men [6].

The free radical theory of aging, which postulates that cumulative oxidative damage inflicted by reactive oxygen species (ROS) drives the aging process, has been a central paradigm in understanding organismal longevity [7]. The idea that oxidative stress plays a central role in aging was first proposed in 1956 by Denham Harman [8]. It was later refined with the discovery of the redox machinery such as the enzyme superoxide dismutase (SOD). This led to the “rate of living” hypothesis, which proposes that energy expenditure is linked to aging through the increased production of ROS via cellular respiration [9]. Although the role of intrinsic factors such as mitochondrial dysfunction and endogenous metabolic processes have received significant attention to understand aging [10], the contribution of extrinsic factors, notably environmental pollutants, has been less thoroughly examined.

Environmental pollutants encompass a wide range of chemical and particulate agents that are ubiquitous [11]. These agents can enter biological systems through inhalation, ingestion, and dermal absorption. Once internalized, many pollutants such as pesticides disrupt cellular redox homeostasis by generating ROS, impairing antioxidant systems, and altering signaling pathways that regulate cellular defense mechanisms [12]. The perturbation of redox balance has downstream effects on mitochondrial integrity, genomic stability, and cellular repair processes—all of which are central to the aging process [13].

The idea that environmental factors contribute to the aging process has been recognized for decades. It has been even proposed that environmental agents can accelerate molecular aging, introducing the term “gerontogen” to describe such toxicants that promote age-related decline [14]. This is still largely ignored and thus a review which synthesizes current knowledge on the types of environmental pollutants that have been implicated in the aging process and details the mechanisms by which these pollutants induce oxidative stress is needed.

## 2. Environmental Pollutants Affect Human Longevity

Human studies provide consistent evidence that exposure to environmental pollutants shortens lifespan. Long-term cohort studies have shown that populations exposed to elevated levels of airborne particulate matter experience increased mortality. Lifelong exposure to particulate matter that has a diameter of 2.5 micrometres or smaller (PM_2.5_) was associated with a global reduction in average life expectancy at birth of approximately one year, with more pronounced decreases of around 1.2 to 1.9 years in highly polluted regions of Asia and Africa [15]. Achieving the World Health Organization’s Air Quality Guideline for PM_2.5_ (10 μg m⁻^3^) across all countries could lead to an estimated population-weighted median increase in life expectancy of 0.6 years [15]. Similar observations have been reported in European cohorts [16].

Heavy metals also demonstrate a clear link with reduced human longevity. Elevated blood lead levels have been shown to correlate with increased cardiovascular disease risk and shortened telomeres in blood [17,18]. The annual costs of lead exposure include 5.5 million premature deaths from cardiovascular disease [19]. Cadmium (Cd) exposure has been associated with renal dysfunction and an increased risk of death [20]. Mercury (Hg) exposure, particularly in communities reliant on fish from contaminated waters, has been linked to neurological impairments and a higher risk of cardiovascular events [21]. This is even corroborated by studies of epigenetic biological aging. Arsenic (As) exposure was linked to advanced biological aging, as indicated by increased GrimAge (an epigenetic clock that estimates biological age) and accelerated DunedinPACE (an epigenetic clock that estimates the pace of aging) DNA methylation scores. This accelerated aging partially mediated the association between arsenic exposure and elevated risks of cardiovascular disease [22]

Organic pollutants, including endocrine-disrupting chemicals, have been the focus of several human studies. Bisphenol A (BPA) exposure, measured in urinary concentrations, has been associated with an increased prevalence of metabolic disorders and cardiovascular diseases [23].

Microplastics are emerging risk factors for chronic diseases. Although the studies on the health risks of different types of plastic particles are only emerging, several association studies have pointed to adverse health outcomes. In a study of 304 patients, the detection of polyethylene in the carotid artery plaque was linked to a higher risk for a myocardial infarction, stroke, or death at 34 months follow-up [24]. In another recent study, the bioaccumulation of microplastics in the brain was linked to a higher risk of dementia diagnosis [25].

Polychlorinated biphenyls (PCBs) have been linked to a range of adverse outcomes. In one study of residents near PCB-contaminated sites, individuals with higher PCB body burden showed a greater incidence of metabolic syndrome and cancer [26,27].

## 3. Exposure to Toxic Chemicals Can Cause Epigenetic Age Acceleration

Epigenetic “clocks” use DNA methylation patterns to estimate biological age and can deviate from chronological age. A positive deviation—epigenetic age acceleration—means the tissue or person is biologically older than expected for their chronological age has been linked to higher mortality risk and earlier onset of age-related disease [28]. Because these clocks capture cumulative molecular changes, they provide a unique toxicogenomic tool to quantify how environmental xenobiotic exposures may accelerate biological aging (Table 1).

Ambient air has consistently been associated with epigenetic aging. A study of elderly participants in the Lothian Birth Cohort (ages 70–80) showed that air pollution around birth and in young-to-middle adulthood was linked to accelerated epigenetic aging and telomere-associated aging in later life [29]. By contrast, prenatal exposures might sometimes produce different effects. Higher exposures to O_3_ and particulate matter in the preconception and prenatal period for two pregnancy cohorts were associated with a deceleration of epigenetic age in newborns [30].

Beyond ambient air pollutants, occupational and indoor airborne toxicants also impact epigenetic clocks. Using a DNA methylation aging marker, a study found that exposure to polycyclic aromatic hydrocarbons (PAHs) was associated with accelerated methylation aging [31]. Benzene and trichloroethylene exposures also increased epigenetic age acceleration in an occupational setting [32]. Exposure to glyphosate and its primary metabolite, aminomethylphosphonic acid (AMPA), was associated with DNA methylation alterations potentially linked to cancer development in 392 postmenopausal women [33]. Notably, the study found that AMPA, but not glyphosate, was associated with increased epigenetic age acceleration. The Agricultural Lung Health Study found that exposure to several currently marketed pesticides and the banned organochlorine heptachlor was associated with differential DNA methylation, with CpGs linked to epigenetic age acceleration for dichlorodiphenyltrichloroethane [34].

Exposure to toxic heavy metals—such as lead (Pb), Cd, As, and Hg—is consistently associated with epigenetic age acceleration in humans. A 2025 analysis of 2200 older adults in NHANES (a representative U.S. sample) found that blood lead and Cd levels were each associated with significantly higher epigenetic ages across multiple clocks [35]. Lead was associated with GrimAge acceleration in a focused study in Detroit, an industrially polluted city, in 290 adults with longitudinal blood samples [36]. Hg exposure correlated with higher PhenoAge acceleration in the same cohort [36]. An extensive study in American Indian communities (Strong Heart Study) where As, Cd, and tungsten (W) exposures are elevated examined metal mixtures and DNAm aging [37]. The combined burden of non-essential toxic metals (As, Cd, W) was positively associated with increased GrimAge acceleration and a faster DunedinPACE [37]. In contrast, the mixture of essential trace elements selenium, zinc, and molybdenum (which the body needs in moderation) was linked to lower biological age—a protective, decelerating effect [37]. There is even evidence from Mendelian randomization that genetically higher iron levels causally increase epigenetic aging [38].

Wildfire-related PM_2.5_ exposure is linked to accelerated epigenetic aging. In a twin and family study, a 1 µg/m^3^ increase in wildfire-related PM_2.5_ was associated with a 0.25-year increase in GrimAge1 acceleration and a 0.36-year increase in GrimAge2 acceleration [39]. 

Early-life exposures can shape future health via accelerated biological aging. In a study of 1173 seven-year-old children from the Human Early-Life Exposome project, more than 100 distinct environmental factors during pregnancy and childhood were examined in relation to epigenetic age acceleration [40]. Associations were found between maternal tobacco smoking during pregnancy and increased age acceleration, while childhood indoor particulate matter absorbance and parental smoking also showed similar trends. In contrast, exposure to the organic pesticide dimethyl dithiophosphate and polychlorinated biphenyl (PCB)-138 appeared protective which also showed that one should not necessarily assume negative impact of synthetic chemicals simply because they are xenobiotics.

Environmental chemicals are not limited to unintentional contaminants; they also include substances that are intentionally ingested, such as alcohol or tobacco. Alcohol intake was linked to accelerated biological aging among middle-aged and older individuals using longitudinal data from cohorts in China and the United Kingdom [41]. A study examining whether epigenetic age acceleration mediates the link between smoking and health outcomes in 2474 Taiwanese participants found that epigenetic clock changes reflected the effects of smoking on diabetes-related outcomes [42]. In a study of 4358 women from the Sister Study [43], researchers found that use of hair permanents/body waves showed a modest link to higher DunedinPACE among all participants and lower PhenoAgeAccel (a metric which quantifies biological aging rate using clinical biomarkers measured in blood) among Black women, but these effects were not consistent across dose or different epigenetic measures [43].

**Figure 1:**
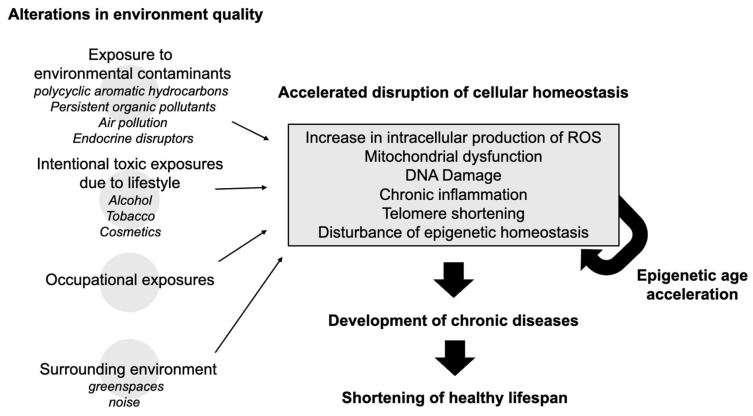
Conceptual framework/overview of how environmental exposure to toxic chemicals can negatively impact healthy longevity.

Recent evidence suggests that environmental quality extends its influence on aging beyond chemical exposures to include features of the broader environment, such as surrounding greenness. Studies have shown that higher levels of nearby vegetation are associated with slower biological aging, as measured by DNA methylation-based clocks like GrimAge [44,45]. This finding expands the scope of environmental health (Figure 1), linking not only pollutant exposure but also the quality of one’s living environment to the pace of biological aging.

## 4. Years of Life in Good Health Lost Due to Environmental Exposures

Environmental factors are estimated by the World Health Organization (WHO) to account for approximately 25% of the total burden of disease globally, which translates into a substantial loss of healthy life years on a population level [46]. Using a back-of-the-envelope calculation based on WHO metrics, one can approximate that environmental exposures result in loss of several years of good health over the lifespan. This environmental burden is quantified using Disability-Adjusted Life Years (DALYs), which represent the total number of years lost due to ill health, disability, or premature death. WHO estimates that approximately 1.7 million DALYs are lost annually in France due to environmental factors [46]. To translate this population-level burden into an individual context, we can estimate annual per capita loss by dividing the annual 1.7 million DALYs by France’s population of approximately 66 million which yields an average loss of about 0.0258 DALYs per person per year. Since one DALY equates to one lost year of healthy life, this corresponds to roughly 9.4 days of healthy life lost per person each year. Over an average lifespan of 80 years, this annual loss accumulates to approximately 2.1 years of healthy life lost per individual due to environmental exposures. This estimation reaches 3–4 years for the most polluted countries like China. This calculation does not take into account interindividual variations and it is likely that individuals who are exposed to pollution levels orders of magnitude higher than others will suffer in proportion.

However, it is important to note that this calculation does not account for other emerging and less-characterized pollutants. Endocrine-disrupting chemicals (EDCs), for instance, are not routinely included in conventional environmental burden assessments despite mounting evidence of their adverse effects on neurodevelopment, reproductive health, and metabolic regulation. Grandjean and Bellanger (2017) suggested that adding functional deficits, especially regarding cognition, to the total environmental burden of disease lead to total costs that are substantially higher than those calculated in terms of the DALY losses that are linked to specific medical diagnoses [47].

Moreover, another underestimated source of lost healthy life years stems from environmental quality degradation associated with climate change. Several studies have shown that climate change exacerbates traditional environmental risks by altering the distribution and intensity of air pollution, increasing the frequency of heatwaves, and influencing the transmission dynamics of infectious diseases. These factors are not captured in standard environmental burden assessments. According to an estimation of the situation in South Korea, the total burden of disease will be 11.48 DALY/1000 population in 2100, which is twice the total burden of disease in 2008 [48].

While the World Health Organization’s estimates suggest that environmental factors account for an average loss of approximately 2–4 years of healthy life per individual, these figures likely underestimate the true impact. When additional contributors—such as endocrine disruptors and the indirect effects of climate change on environmental quality—are incorporated, the cumulative loss of healthy life years could conceivably reach between 5 and 10 years per person.

**Table 1:** Key studies showing acceleration or deceleration of epigenetic age after exposure to environmental pollutants.

Study (Year)	Exposure(s)	Study Type	Key Findings on Age Acceleration
Ward-Caviness et al., 2016 [49]	Chronic ambient air pollution to PM_10_, PM_2.5_, PM_2.5_ absorbance/black carbon (BC), and NOx	Cohort (older adults)	A 0.97 µg/m^3^ increase in PM_2.5_ was linked to a 0.33-year increase in extrinsic epigenetic age acceleration (95% CI = 0.01–0.64; *p* = 0.04). BC and NOx exposures were associated with DNAmAA and intrinsic epigenetic age acceleration in women. Telomere length (TeloAA) was inversely associated with BC in men. A multiple phenotype analysis found BC and PM_10_ to be broadly associated with biological aging in men.
de Prado-Bert et al., 2021 [40]	Early-life tobacco smoke and indoor PM	Multi-cohort (age ~7)	Prenatal smoking and high childhood PM exposure accelerated epigenetic age (Horvath’s Skin and Blood clock) in children. Dimethyl dithiophosphate and PCB 138 were protective.
van der Laan et al., 2022 [32]	Occupational solvents (benzene, TCE, formaldehyde)	Cross-sectional (workers)	Occupational benzene and TCE exposure were associated with increased epigenetic age acceleration measured by the Skin and Blood Clock.
Song et al., 2022 [30]	Prenatal ambient air pollution (PM2.5, O₃)	Cohort (birth outcomes)	Higher air pollution exposure during preconception and pregnancy was linked to decelerated epigenetic age at birth (newborn clocks). Suggests altered fetal epigenetic development from pollution.
Shi et al., 2022 [50]	Personal airborne chemicals (including phthalates)	Cross-sectional (older adults)	Phthalate exposure showed significant acceleration of DNAm PhenoAge. In 60–69 y olds, those with higher phthalate and VOC exposure had higher phenotypic epigenetic age.
Boyer et al., 2023 [37]	Metal mixture: arsenic (As), cadmium (Cd), tungsten (W) (plus essential metals)	Cross-sectional (American Indian cohort)	Toxic metal mixture (As, Cd, W) was associated with higher GrimAge acceleration and faster DunedinPACE (aged faster biologically). Essential metals (Se, Zn, Mo) were linked to lower epigenetic age (protective). Cd had the strongest pro-aging effect
Lodge et al., 2022 [36]	Lead (Pb), mercury (Hg), manganese (Mn), copper (Cu)	Longitudinal cohort (polluted city)	Pb exposure → higher GrimAge age. Hg → higher PhenoAge. Mn was inversely associated with PhenoAge (potentially slower aging). Cu showed a U-shaped relationship. Overall metal mixture → GrimAge and PhenoAge acceleration increased, Horvath age paradoxically decreased.
Choi et al., 2025 [51]	TCDD dioxin (high vs. low exposure)	Cross-sectional (older adults)	Dioxin (TCDD) exposure was associated with accelerated aging, especially on mortality-predictive clocks. Higher TCDD dose → higher GrimAge and PhenoAge acceleration (dose–response). Identified TCDD as a potent environmental aging accelerator.
Goodrich et al., 2021 [52]	Per- and polyfluoroalkyl substances (PFAS)	Cross-sectional (firefighters)	No significant positive epigenetic age acceleration from PFAS exposure. Most PFAS showed no effect on DNAm age. Notably, PFDA and PFUnDA were linked to lower GrimAge (inverse association). Overall, PFAS did not measurably accelerate epigenetic aging in this high-exposure occupational group.
Hoang et al., 2021 [34]	Pesticide exposure	The Agricultural Lung Health Study nested within the Agricultural Health Study	A total of 162 differentially methylated CpGs were identified for nine pesticides (eight current, one banned). CpGs were unique to each active ingredient and often showed dose–response relationships. 28% were linked to cis-gene expression, suggesting functional effects. A previously reported association between DDT exposure and epigenetic age acceleration was confirmed.
Lucia et al., 2022 [33]	Glyphosate and its metabolite aminomethylphosphonic acid (AMPA)	Postmenopausal women residing in southern California between the ages of 45 and 66 y (*N* = 392)	AMPA, but not glyphosate, was associated with greater epigenetic age acceleration.

## 5. Mechanistic Linking Pollutant-Induced Oxidative Stress and Biological Aging

Environmental pollutants induce oxidative stress through multiple, interrelated pathways. The mechanisms described below outline how these agents disrupt redox balance and contribute to cellular damage.

### 5.1. Redox Imbalance and ROS Generation

Many environmental pollutants exert their toxic effects by directly or indirectly increasing the intracellular production of ROS. Heavy metals, for instance, can participate in redox cycling reactions [53]. The Fenton reaction, which involves transition metals such as iron, is enhanced by pollutants that disturb metal homeostasis. Although heavy metals like cadmium do not directly catalyze Fenton reactions, they induce oxidative stress by depleting cellular antioxidants and impairing repair systems [54].

Several key signaling pathways are modulated by pollutant-induced oxidative stress. The nuclear factor erythroid 2–related factor 2 (Nrf2) is a transcription factor that regulates the expression of antioxidant and detoxification genes [55]. Under conditions of oxidative stress, Nrf2 is released from its inhibitor Keap1 and translocates to the nucleus. Exposure to polychlorinated biphenyl quinone leads to persistent Nrf2 activation, which may result in compensatory mechanisms that are insufficient to counterbalance continuous ROS production [56].

### 5.2. Biotransformation via Cytochrome P450 Enzymes

Organic pollutants may undergo biotransformation via cytochrome P450 enzymes. This metabolic activation often results in the formation of reactive intermediates capable of donating electrons to molecular oxygen, thereby generating ROS [57]. Hepatic microsomal enzymes metabolize lower chlorinated polychlorinated biphenyls (PCB) (mono-, di-, and trichlorobiphenyl congeners) to hydroxylated derivatives, notably catechols and hydroquinones [58]. These metabolites are redox-active and can undergo one-electron oxidation or reduction, forming semiquinone radicals that participate in redox cycling, leading to the generation of ROS [59]. In parallel, PCB biotransformation may yield electrophilic intermediates such as arene oxides and quinones, which can form covalent adducts with nucleophilic sites on DNA, proteins, and other cellular macromolecules, contributing to PCB-mediated genotoxicity [60].

### 5.3. Mitochondrial Dysfunction

Mitochondria are central to cellular energy production and are a primary site of ROS generation. Mitochondrial dysfunction has been reported to be associated with cellular senescence, which in turn contributes to aging [61,62,63]. Exposure to environmental pollutants has been associated with mitochondrial dysfunction through several mechanisms. Heavy metals can interfere with components of the mitochondrial electron transport chain, resulting in electron leakage and the formation of superoxide radicals [64]. For instance, the pesticide paraquat exerts its toxicity primarily by generating superoxide radicals through mitochondrial redox cycling leading to oxidative stress which collectively contribute to Parkinson’s disease [65].

### 5.4. DNA Damage, Protein Oxidation, and Lipid Peroxidation

The ROS generated in response to pollutant exposure can inflict damage on critical cellular macromolecules. Oxidative modifications of nucleic acids result in lesions such as 8-oxoguanine, which can lead to mutations if left unrepaired [66]. Arsenic-induced DNA damage is further compounded by the inhibition of DNA repair enzymes [67]. Proteins, particularly those with redox-sensitive cysteine residues, are vulnerable to oxidation [68]. The resulting structural changes may impair enzyme activity and protein–protein interactions.

Lipid peroxidation is another major consequence of ROS overproduction. The oxidation of polyunsaturated fatty acids in cellular membranes results in the formation of reactive aldehydes, such as 4-hydroxynonenal (4-HNE) [69]. These secondary products can form adducts with proteins and nucleic acids, exacerbating cellular dysfunction and contributing to the propagation of oxidative damage.

### 5.5. Inflammatory Responses

Aging is characterized by systemic chronic inflammation [70]. Exposure to environmental pollutants often initiates inflammatory responses that can be initiated by oxidative stress or further amplified by oxidative stress. Particulate matter and certain organic compounds can activate resident immune cells, including alveolar macrophages, resulting in the release of pro-inflammatory cytokines such as interleukin-6 (IL-6) and tumor necrosis factor-α (TNF-α) [71]. The chronic nature of this inflammatory milieu leads to the persistent activation of NADPH oxidases which drives hippocampal dysfunction in experimental multiple sclerosis [72]. Oxidative stress is the molecular initiating event, driving inflammation and toxicity across biological levels after exposure to micro- and nanoplastics [73].

### 5.6. Telomere Dynamics

The rate of increase in short telomeres is a predictor of longevity in mammals [74]. ROS can accelerate telomere shortening by inducing single-strand breaks and interfering with the activity of telomerase, the enzyme responsible for telomere elongation. Out of 22 independent experiments reported from seven different laboratories, mild oxidative stress accelerated telomere shortening in all but three experiments, and antioxidants slowed it and extended cell lifespan [75]. Exposure to airborne particulate matter has been correlated with reduced telomere length in epidemiological studies [76]. The accelerated telomere attrition observed in populations exposed to high levels of environmental pollutants suggests a direct mechanistic link between external toxicants and cellular replicative capacity even if this is much less important mechanistically than the increased oxidative stress in promoting telomere shortening. This effect is compounded by the fact that shortened telomeres predispose cells to genomic instability and senescence, thereby contributing to shortening healthy lifespan.

### 5.7. Autophagy and Proteostasis

Autophagy is a cellular process responsible for the degradation and recycling of damaged organelles and proteins [77]. Proper autophagic function is essential for maintaining proteostasis, a key determinant of cellular longevity. Furthermore, the inhibition of autophagy or the depletion of its resources can either exacerbate or mitigate toxicity, contingent upon the specific context [78]. Selective autophagy is a critical cellular process in mammalian systems, facilitating the targeted removal of particulate matter, nanoparticles, toxic metals, and components resulting from smoke exposure, while preserving the integrity of cytosolic structures [79]. For instance, exposure to cadmium has been associated with the disruption of autophagosome formation in the development of neurodegenerative disorders [80], while perfluorooctane sulfonate induces autophagy-dependent lysosomal membrane permeabilization that can alter lysosomal function and induce hepatoxicity [81]. Impaired autophagy results in the accumulation of dysfunctional cellular components, thereby exacerbating oxidative stress and promoting cellular aging. Altered autophagy phenotypes are linked to lung diseases such as chronic obstructive lung disease, acute lung injury, cystic fibrosis, idiopathic pulmonary fibrosis, pulmonary arterial hypertension, and asthma [82].

### 5.8. Linking Oxidative Stress with Epigenetic Aging

Nutritional factors and environmental exposures converge on epigenetic mechanisms via oxidative stress. For example, oxidative stress can lead to DNA hypomethylation or hypermethylation by affecting the activity of DNA methyltransferases (DNMTs) and ten-eleven translocation (TET) enzymes, thereby changing the methylation status of genes involved in antioxidant defense such as after prenatal and ancestral exposure to di(2-ethylhexyl) phthalate [83]. Similarly, ROS can modify histone proteins through altered acetylation, which impact chromatin structure and gene expression [84]. Dietary phytochemicals such as curcumin and resveratrol have been shown to modulate the activity of epigenetic regulators like histone acetyltransferases (HATs) and histone deacetylases (HDACs), offering protection against oxidative stress-induced epigenetic alterations [85]. Hypermethylation of antioxidant genes can reduce their expression, leading to increased ROS accumulation [86,87,88]. These epigenetic modifications can change the rate at which the damages caused by oxidative stress accumulate, thereby influencing biological age by affecting cellular repair processes and the overall resilience of the organism.

## 6. Implications for Aging and Longevity

Environmental pollutants constitute an often-overlooked factor in the aging process. The mechanistic insights presented in this manuscript provide a snapshot of how specific classes of pollutants—including heavy metals, particulate matter, and endocrine-disrupting chemicals—induce oxidative stress through multiple pathways. These pollutants disrupt redox balance, impair mitochondrial function, and damage critical biomolecules such as DNA, proteins, and lipids, ultimately affects epigenetic aging.

The cumulative impact of these events has significant implications for the overall trajectory of organismal aging. Epidemiological evidence linking pollutant exposure to cardiovascular, neurodegenerative, and oncologic outcomes further supports the concept that environmental health is an integral component of the longevity equation. In a recent study comparing genetic and environmental influences for 22 major diseases, polygenic risk scores explained less than 2 percentage points of additional mortality variation, whereas the exposome explained an additional 17 percentage points [89].

Beyond their direct biological impact, many environmental pollutants originate from unsustainable consumption patterns and waste accumulation associated with modern industrialized lifestyles. Reducing exposure to these pollutants therefore not only supports public health, but also aligns with broader goals of environmental sustainability [90].

Addressing the challenge posed by environmental pollutants requires coordinated efforts at multiple levels. Regulatory measures aimed at reducing ambient pollutant levels, combined with lifestyle modifications and targeted therapeutic interventions, may collectively mitigate the adverse effects of these toxicants on cellular and systemic aging processes. 

## 7. Conclusions

The incorporation of environmental factors into aging research not only expands the conventional paradigm but also highlights the importance of a holistic approach to promote healthy aging. By recognizing and addressing the contributions of environmental pollutants to oxidative stress, the scientific community can advance strategies that extend healthspan and improve quality of life in aging populations.
